# *In vivo* X-Ray Computed Tomographic Imaging of Soft Tissue with Native, Intravenous, or Oral Contrast

**DOI:** 10.3390/s130606957

**Published:** 2013-05-27

**Authors:** Connor A. Wathen, Nathan Foje, Tony van Avermaete, Bernadette Miramontes, Sarah E. Chapaman, Todd A. Sasser, Raghuraman Kannan, Steven Gerstler, W. Matthew Leevy

**Affiliations:** 1 Department of Biological Sciences, 100 Galvin Life Sciences Center, University of Notre Dame, Notre Dame, IN 46556, USA; E-Mail: cwathen@nd.edu; 2 Department of Chemistry and Biochemistry, 236 Nieuwland Science Hall, University of Notre Dame, Notre Dame, IN 46556, USA; E-Mails: nfoje@nd.edu (N.F.); tonyvan22@gmail.com (T.V.A.); bmiramontes25@phm.k12.in.us (B.M.); todd.sasser@carestream.com (T.A.S.); 3 Penn High School, 55900 Bittersweet Road, Mishawaka, IN 46545, USA; 4 Notre Dame Integrated Imaging Facility, Notre Dame, IN 46556, USA; E-Mail: sarah.chapman55@gmail.com; 5 Bruker-Biospin Corporation, 4 Research Drive, Woodbridge, CT 06525, USA; 6 Department of Radiology, University of Missouri, Columbia, MO 65212, USA; E-Mail: kannanr@health.missouri.edu; 7 Saint Joseph Regional Medical Center, Mishawaka, IN 46545, USA; E-Mail: sgerstler@yahoo.com; 8 Harper Cancer Research Institute, A200 Harper Hall, Notre Dame, IN 46530, USA

**Keywords:** X-ray CT, computed tomography, X-ray contrast agents, review, lungs, adipose, gi tract, vasculature, kidney, liver

## Abstract

X-ray Computed Tomography (CT) is one of the most commonly utilized anatomical imaging modalities for both research and clinical purposes. CT combines high-resolution, three-dimensional data with relatively fast acquisition to provide a solid platform for non-invasive human or specimen imaging. The primary limitation of CT is its inability to distinguish many soft tissues based on native contrast. While bone has high contrast within a CT image due to its material density from calcium phosphate, soft tissue is less dense and many are homogenous in density. This presents a challenge in distinguishing one type of soft tissue from another. A couple exceptions include the lungs as well as fat, both of which have unique densities owing to the presence of air or bulk hydrocarbons, respectively. In order to facilitate X-ray CT imaging of other structures, a range of contrast agents have been developed to selectively identify and visualize the anatomical properties of individual tissues. Most agents incorporate atoms like iodine, gold, or barium because of their ability to absorb X-rays, and thus impart contrast to a given organ system. Here we review the strategies available to visualize lung, fat, brain, kidney, liver, spleen, vasculature, gastrointestinal tract, and liver tissues of living mice using either innate contrast, or commercial injectable or ingestible agents with selective perfusion. Further, we demonstrate how each of these approaches will facilitate the non-invasive, longitudinal, *in vivo* imaging of pre-clinical disease models at each anatomical site.

## 1. Introduction

X-ray computed tomography (CT) was developed in the 1970s by Sir Godfrey Hounsfield and Allan MacLeod Cormack, and has become a critical diagnostic and imaging tool in both research and clinical settings [1]. The technology works by acquiring planar X-ray images (or projections) taken at various degrees of rotation around a patient or specimen. These data are then reconstructed, typically with a filtered back projection algorithm, to produce a three-dimensional array of radiodensity values. Frequently, these data are calibrated to Hounsfield units (HU) which indicate radiodensity, and are calculated using the equation below:

$$\text{HU}=1000\times {(\mu }_{\text{x}}-\mu_{\text{water}}{)/\mu }_{\text{water}}$$

The calibrated Hounsfield scale will have values of -1,000 HU to represent air, 0 HU to denote water, and up to 3,000 HU for dense bone. Soft tissues, which are primarily composed of water and protein, will have densities in the +100 to +300 range [2] and can be particularly difficult to differentiate via CT due to their low radio-opacity.

There are several advantages to the use of X-ray CT for anatomical imaging. First, the tomographic data can be obtained with relative speed and ease. A typical scan is completed in 0.5–5 minutes using equipment that is ubiquitously available at hospitals and most research institutions. For the pre-clinical scientist, there are a number of convenient and free software packages available for analysis and rendering of the CT data; for example ImageJ (NIH) [3], Volview (Kitware Inc), and Amide [4] to name a few. Next, CT imaging is non-invasive and, under the appropriate settings, the dose of radiation received from the scan will not harm, alter, or otherwise interfere with the biochemistry or life cycle of subjects [5,6]. Perhaps the most important benefit of the X-ray CT modality is the high-resolution data that are collected. Since clinical scanners are examining relatively large anatomical structures within patients, a 1 mm [7] resolution is sufficient for diagnosis. However, this must be dramatically improved for pre-clinical research due to the much smaller size of rat or mouse anatomy. These scanners are typically in the 30–300 µm range and are sometimes called microCT. With appropriate attention to acquisition parameters, this resolution can be achieved with radiation doses below critical biological thresholds, even with multiple scans during longitudinal imaging studies. Therefore, non-invasive microCT imaging provides an excellent tool for longitudinal and comparative studies within the bone and soft tissues of living mice. *Ex vivo* specimen platforms are also available to achieve resolution as fine as 1 µm, but may not be used on living specimens due to radiation hazard from collecting additional projections with high geometric magnification.

Within the anatomical imaging space, X-ray CT is commonly compared to magnetic resonance imaging (MRI) since both modalities are non-invasive, and provide a volumetric output for analysis and visualization. In the case of CT, the output is a three-dimensional array of radiodensity values, while MRI produces a matrix of values related to water proton relaxation time within a magnetic field and after a radiofrequency pulse. One of the primary clinical differences between CT and MRI is that the former is generally used for imaging dense structures like bones, while the latter is typically better at resolving soft tissues [8]. For pre-clinical research in particular, the use of MRI is hindered by significantly longer acquisition times to achieve similar levels of resolution, thus reducing throughput [9]. Further, the availability of small animal MRI systems is generally restricted to larger medical research institutions. Finally, MRI requires an increased level of technical expertise to utilize the instrumentation relative to X-ray CT, which will compound the obstacles that general pre-clinical researchers face in accessing the technology. However, new developments in benchtop MRI systems are helping to reduce the technical and accessibility barriers of this equipment to stimulate usage among the broader pre-clinical and biological research disciplines [10]. In this review we focus on the methods and reagents available to make X-ray CT compatible with *in vivo* soft tissue imaging in a pre-clinical setting.

When planar X-ray images are acquired, tissues and other materials will attenuate X-rays based upon their own unique radio-opacity and composition. In general, structures that are denser and contain large quantities of electron rich elements absorb greater amounts of X-rays, while less dense materials absorb smaller amounts [2]. The contrast noted in the planar X-ray projections will translate to the computed tomography data to enable the visualization of several types of tissue using the innate properties of the anatomy under observation. Bone, which is radio-dense and rich in calcium, absorbs large amounts of X-ray radiation and provides the highest natural X-ray attenuation. Lung tissue is distinguishable owing to the presence of air within its cavity, thus yielding significantly lower densities than the surrounding soft tissue and bone [2]. Adipose is high in fatty acids and is slightly less dense than adjacent soft tissue, which enables its recognition via segmentation with the use of the proper software [11]. Finally, brain tissue is generally homogenous on a CT, but its location is circumscribed by cranial bone and the tissue may be directly observed for changes occurring during disease progression [12,13].

X-ray contrast agents are injectable or ingestible compounds with high electron density that will attenuate X-rays. Many contrast agents will perfuse or preferentially concentrate at a specific tissue, thus enabling its visualization and differentiation from surrounding sites. There are several broad categories of contrast agents. The most widely used group utilizes molecules that incorporate multiple iodine atoms while maintaining water solubility and biocompatibility. Prominent examples of intravenous contrast agents include the commercially available Visipaque™ [14] and Omnipaque™ [15]. The most commonly employed P.O. (oral) contrast agent is barium sulfate, which is biologically inert and can be safely ingested to enhance visualization of the digestive tract [16]. Recent research has focused on nanoparticles, specifically gold and alkaline earth metals, as novel contrast enhancing agents for several types of soft tissue. With the aid of these contrast agents, visualization of specific organs is greatly improved, which consequently leads to recognition of anatomical abnormalities, and in some cases, functional changes in rates of perfusion or clearance during studies of disease progression.

Recent advances in imaging technology, contrast agent development, and analytical software have vastly increased the number of potential applications of CT imaging. Here we review the current applications and contrast agents available for pre-clinical X-ray microCT imaging of soft tissue in mice and rats. This review is organized by individual anatomical location, with example *in vivo* microCT images from mice provided by our laboratory. Each image we present was reconstructed at a 125 µm isotropic voxel size, which represents an intermediate level of resolution that balances a wider field of view (65 mm) with a moderate data size (583 MB) and reasonable radiation dose (deep dose equivalent = 220 mGv for single scan on the Albira Image Station) that is approximately 1%–2% of the LD 50/30 (50% mortality within 30 days) [17]. These data are presented in the form of the classic sagittal, coronal, and transverse/axial greyscale slices that are common among different scanners and research groups. We also provide a facile 3D rendering of each data set purely as an example derived from a freeware software program, and note these types of display are highly variable in the literature, and the “quality”of these types of visualizations is often subjective. Nevertheless, this format was chosen to ensure continuity among the various figures at different anatomical locations, and note that experimental conditions may be optimized to interrogate given structures with higher resolution at smaller fields of view. Each example was collected using a pre-clinical microCT in conjunction with commercially available contrast media to highlight the experiments for which a researcher will have immediate access. The methods for animal preparation, *in vivo* image acquisition, analysis, and visualization are also provided.

## 2. Methods

For imaging native contrast (*i.e.*, lung, fat, and brain), animals received no contrast agent prior to imaging. For imaging of vasculature, liver, kidneys and GI tract animals received contrast agent via retroorbital or *per os* administration (see Table 1 below). Imaging was performed using the Albira PET-SPECT-CT imaging station (Bruker Biospin Corp., Woodbridge, CT, USA). The Albira CT system was factory calibrated and provided the imaging data output in Hounsfield units (HU). For all X-ray CT imaging, mice were anesthetized with 3% isoflourane then immediately placed in the animal bed within the instrument, where anesthesia was maintained via nose cone. CT scans were acquired at 45 kV with a 400 µA current, and 600 projections collected to yield a 125 µm voxel size after reconstruction via filtered back projection. Imaging was performed with either a 118.5 mm or 65.0 mm axial FOV. After reconstruction with the Albira software, the slice data was viewed and analyzed with PMOD v3.17 (PMOD Technologies Ltd., Zurich, Switzerland).

For slice view displays, the data was typically contrasted to between −200 and 500 HU and a jpeg file captured using PMOD software in which the transverse, coronal and sagittal slices were displayed. PMOD was subsequently used for all masking and segmentation to generate anatomy specific data sets. Segmentation for lung and fat tissue was applied using HU ranges characteristic of these tissues after masking some image features. For animals receiving contrast agents, volumes of interest (VOIs) were manually drawn around target structures, with subsequent masking of the external VOI space to create an independent data set.

These images were subsequently overlaid on the original CT images in the 3D display software VolView (v 3.4 Kitware Inc.). A range of color palettes were chosen for the target anatomy to illustrate a couple of the common intensity schemes for 3D rendered data. ImageJ v 1.43 u software was then used to create a montage of the three planar images obtained from PMOD along with the 3D rendering obtained from Volview. Figure labels were added using PowerPoint 2007 (Microsoft, Redmond, WA, USA).

## 3. Native Contrast

### 3.1. Lungs

#### 3.1.1. Overview

Lung disease is both common and fatal. In particular, the lung has the highest associated mortality for cancer malignancy with 205,874 new cases recorded in America in 2009, and 158,081 deaths [18]. Other chronic lower respiratory diseases, excluding asthma, were attributed to over 133,000 deaths in 2010, according to the most recent data available from the CDC [19]. X-ray CT is particularly well suited to image the respiratory system due to the uniquely low density within the lung space. In this case, visualization and subsequent quantification of lung air volume may be accomplished without contrast enhancement. The large differences in density between lung and surrounding tissues, such as heart and liver, enable the longitudinal examination of various lung malignancies in small animals. It is possible to quantify changes in lung air volume and morphology over a period of months in order to evaluate the long-term effectiveness of various treatments or progression of untreated diseases like emphysema [20]. Although histological analysis of lung tissue is still the most effective method for detecting emphysema, microCT provides comparative results and thus enables longitudinal experiments [20]. MicroCT also achieves high enough resolution to enable imaging of single alveoli during terminal end point studies of this disease model [21]. In the case of fibrosis, lesions may be observed and similar lung degradation may be quantified [22]. Another example includes non-invasive monitoring of lung volume degradation due to cancer proliferation. During lung cancer metastasis studies, the air space will be filled with solid tumor tissue that will reduce the volume detected through computational segmentation [23]. MicroCT is useful for detecting numerous types of cancer in the airway, although less effective at detecting tumors around the periphery of the lungs and serosa [24]. In a study comparing ^3^He-MRI to microCT for imaging of the lungs, there was a strong correlation between the measured lung volume obtained by each modality, suggesting that both provide adequate avenues for longitudinal studies measuring lung volume [25].

#### 3.1.2. Example Data and Discussion

**Figure 1.**
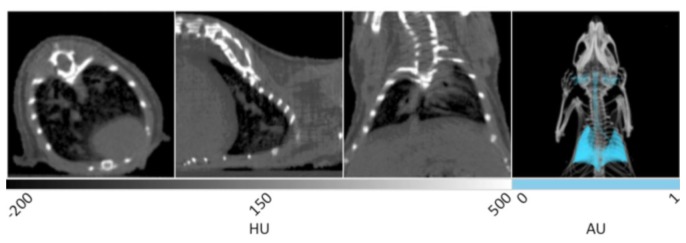
X-ray CT data of murine lungs. The first three panels display transverse, sagittal, and coronal slices of healthy lung tissue. The lung air space is noted as darker volumes due to its low density relative to the surrounding tissue. The fourth panel is a 3D rendering of the same data with the lungs false colored cyan to aid visualization.

Representative *in vivo* X-ray CT slices of healthy murine lung tissue are displayed in Figure 1. From left to right, the first three frames show the transverse, sagittal, and coronal planes respectively, and are windowed between -200 and 500 HU for optimal display of bone, lungs, and surrounding tissue. This is a greyscale display of the imaging data in which areas of low density appear darker, while areas of high density appear white. In the transverse plane, the spinal column, rib cage, and sternum are noted as white structures, while the heart is observed as a gray, less defined tissue residing at the bottom right. The very low-density air space within the lung tissue can be seen as the dark region inside the ribcage in each of the three slices. It is within the dark black landscape of the lung air space that the pathogenicity of several disease models may be observed and quantitatively measured in units of cm^3^. The gray tissue within the body of the lung air space shows the branching of pulmonary vasculature. The sagittal plane shows the right lobe of the lungs with similar branching of the airways seen in gray. The coronal and transverse planes display both lobes of the lungs in addition to the spinal column and rib cage.

The fourth panel of Figure 1 shows a 3D rendering of the same data set. The lungs have been segmented using PMOD software in order to provide enhanced visualization. Segmentation allows the isolation of voxels in a predetermined range of Hounsfield Units. Using this tool within the PMOD software, all of the voxels corresponding to lung air volume within the range of −500 to −250 HU can be automatically selected and allocated into a new data set with identical geometry to the parent data to preserve 3D fusion. Once selected, the lung air volume can then be quantified and saved as a unique data set in which the intensity values have been set to 1 or 0 representing positive or negative segmentation. After false coloring of the lung volume data set, the original CT image can be overlaid to provide spatial reference. In the 3D rendering, the entirety of the lungs can be seen in addition to the bronchi, trachea, and ear canals. This rendering enables the visualization of anatomical changes occuring throughout the entire tissue during disease progression, especially those that result in decreased lung volume. Advanced algorithms are currently available to improve the resolution of lung segmentation with disease progression in both clinical [26] and pre-clinical [27] settings.

### 3.2. Adipose Tissue

#### 3.2.1. Overview

According to the Centers for Disease Control and Prevention (CDC), over 1/3 of the United States is clinically obese. Further, over 50% of Americans and Europeans are classified as overweight or obese by the World Health Organization (WHO). Obesity increases the risk for several deadly disorders including heart disease, diabetes, and stroke, so understanding the risk factors and best modes of treatment for this disease are essential [28]. The prevalence of this disease, coupled to the serious health consequences it poses, has placed an intense focus on obesity-related research. Mouse models of obesity are highly prevalent and provide a convenient experimental system with which to study progression of the disease [29]. Fortunately, adipose is one of the few soft tissues that is readily distinguishable in non-contrast enhanced CT images due to the significantly lower material density of fat in relation to other soft tissues [30]. Thus, CT is an excellent modality to conduct longitudinal studies examining the genetic and environmental factors contributing to fat gain or loss using mouse models. Researchers have demonstrated that microCT is an effective tool for the detection and quantification of several types of fat, including subcutaneous, visceral, brown, and intrahepatic fat, each of which has a unique role in obesity disease progression [31,32]. Compared to MRI, X-ray CT is more cost-effective, with higher resolution and commercial availability [33]. Other studies have shown the feasibility of using the differences in tissue densities to segment and quantify adipose, lean, and skeletal tissues [34]. MicroCT computed fat volumes have been shown to closely correlate with values gathered during *ex vivo* analysis [35].

#### 3.2.2. Example Data and Discussion

**Figure 2.**
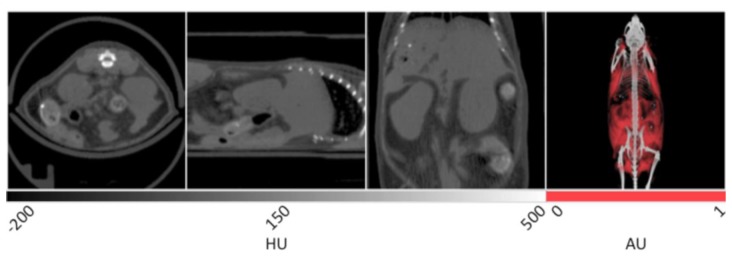
X-ray CT data of murine adipose tissue. In the first three panels, the visceral adipose tissue can be distinguished from the soft tissue of the kidneys and intestines due to its lower density values. In the 3D rendering at right, the spatial distribution of the total fat content can be observed with the majority residing around the abdomen of the mouse.

The slice data and a 3D reconstruction of an obese mouse (B6.V-Lep°^B^/J, male, 12 weeks old) are displayed in Figure 2. In the transverse view shown on the left, significant visceral fat deposits can be observed around the kidneys as lower density volumes owing to the decreased attenuation of adipose. In the sagittal view, more extensive subcutaneous fat deposits can be observed in addition to those in the abdomen. In the coronal view, fat deposits are seen around the kidneys, gastrointestinal tract and liver. The fat volume was segmented in a similar manner as the lungs using PMOD software, however values were bounded to −200 and −50 HU. Once the adipose tissue was segmented and allocated into an independent data set, it was false colored red and overlaid on the original CT to aid visualization. In the right frame of Figure 2, the 3D rendering displays the full extent of the spatial distribution of the fat deposits. Specifically, the extent of the visceral and subcutaneous adipose is highlighted, especially around the limbs and gut area. The segmented adipose tissue may be quantified in units of cm^3^, and divided by the whole animal volume to give a percent body fat value that may be monitored over time and under different experimental conditions.

### 3.3. Brain

#### 3.3.1. Overview

Brain disorders are debilitating and in many cases lethal. Stroke is a particularly morbid disease and is the number four cause of death in the United States [36]. Mouse models have been developed for a range of human brain diseases including cerebral ischemia [37] and Alzheimer's disease [38]. These models provide information about both the progression of these diseases and the effectiveness of experimental treatments. A lack of significant contrast often poses a barrier to the imaging of these disease models with non-contrasted CT. However, since the brain resides in a cavity that is circumscribed by the cranial bones of the skull, it is straightforward to locate. Density changes (ΔHU) that occur with disease progression may be readily measured using VOIs constructed within the brain space. In one prominent example, a rat model of cerebral ischemia was developed in which the right middle cerebral artery was occluded. Cerebral volume density changes in HU were measured before and after the blockage event [39]. This allowed for the monitoring of the edema process, which is analogous to routine use of non-contrasted X-ray CT to diagnose stroke in clinical settings [40].

#### 3.3.2. Example Data and Discussion

**Figure 3.**
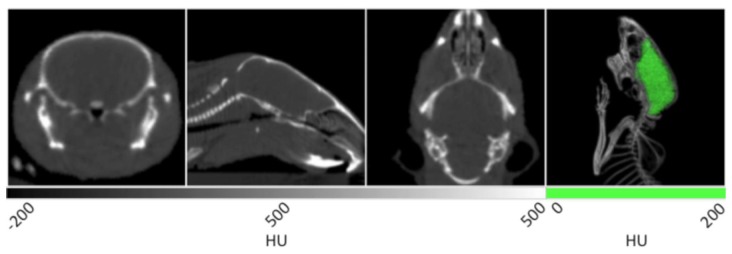
X-ray CT data of a murine brain and skull. Transverse, sagittal and coronal slices are displayed in the first three panels respectively. In the fourth panel is a 3D rendering of the same data with the brain tissue displayed in green.

Without contrast enhancement, the brain can be distinguished from the cranium due to the stark differences in density between the tissue and surrounding bone. The first three panels of Figure 3 show the homogenous grey matter delineated by cranial bone. These frames also display many additional anatomical features. In the transverse slice at left, the trachea is seen as black due to negative contrast at the center of the frame. In the next image showing the coronal view, a larger portion of the trachea can be observed in addition to the nasal cavity and spinal cord. In the 3D rendering at right, the cranium is bisected to clearly show the brain tissue circumscribed by the skull. Because the cranium delineates the brain cavity, it enabled the manual segmentation of brain tissue. Manual segmentation refers to the creation of a VOI by manually drawing a region of interest (ROI) around each slice of the reconstructed brain data. After the VOI was constructed, the data was masked outside of it to isolate the brain tissue and display it in green within the context of the rendered animal CT. As noted above, the density values within the brain may be measured as a whole or in a region specific manner in different lobes.

## 4. Injectable Contrast

### 4.1. Vasculature

#### 4.1.1. Overview

Abnormalities of the cardiovascular system are implicated in several diseases. Abdominal aortic aneurysm (AAA) is one area of particular concern due to the high mortality rate associated with a rupture [41]. Many mouse models of AAA exist, the study of which has the potential to improve the understanding and treatment of the disease [42]. The murine aortic arch has proven to be a valid alternative for studying the human aorta [43]. Tumor angiogenesis in mouse models of cancer are also an active area of study [44]. As noted above, mouse models of cerebral ischemia are extremely important for pre-clinical research of stroke. The similarities between the cardiovascular systems of humans and mice make them valuable disease models for non-invasive imaging with microCT [45].

Imaging with X-ray computed tomography provides a tool to explore vasculature with high fidelity and resolution. However, the native properties of these structures do not permit their differentiation, and thus contrast agents must be used to facilitate their direct observation. For example, the detection of angiogenesis in tumor models is enabled by a bolus injection of an iodinated contrast agent such as Iomeprol 400 immediately prior to microCT imaging [46]. Another vigorous area of vascular research utilizes CT with continuous intravenous infusion of iodinated contrast media like Iopromide 300 to visualize vasculature in the brain. These methods are capable of detecting aneurysms and other precursors to cerebral hemorrhage in stunning detail [47]. One widely used contrast agent is iohexol, which is sold under the name Omnipaque™ by GE Healthcare. Iohexol is an iodinated, non-ionic, water-soluble contrast media available with iodine concentrations of 240, 300, or 350 mgI/mL. Iohexol's applications are often limited by its rapid clearance through the kidneys [48], and is typically used for blood pool imaging in the clinic, in addition to imaging of the liver [49]. In a pre-clinical setting, it has been utilized in mice as an agent for continuous perfusion with a syringe pump during microCT scanning. In this fashion, it provides substantial contrast to cerebral vasculature and is a powerful tool for imaging cerebral occlusions and ischemia [50,51]. With proper catheterization, syringe pump setup, and experimental technique including rapid CT acquisition, the use of iodinated contrast agents will provide excellent contrast to vasculature during fast pre-clinical X-ray CT scans of mice.

For *ex vivo* analysis of cerebral vasculature with the highest possible resolution, a silicon-based contrast agent named Microfil^®^ can be used for vascular casting and subsequent CT scanning with increased geometric magnification and additional projections [52–54]. These settings would generate a radiation dose too high for *in vivo* imaging, but provide an excellent mean for capturing additional detail without further perturbing the vascular architecture of a given organ system.

Gold nanomaterials are a diverse new field of reagents with numerous potential applications, especially within microCT. Among the elements with higher atomic number, gold is relatively non-toxic and possesses excellent X-ray absorption properties (atomic number: 79; K-edge: 80.7 keV), which are pivotal in developing site-specific CT contrast agents. Nanosized gold provides an inherent advantage toward increasing X-ray sensitivity. Each gold nanoparticle (AuNP) contains hundreds of atoms that facilitate X-ray attenuation. Surface atoms can be readily conjugated with target specific biomolecules, such as tumor-avid antibody, and for site-specific delivery of contrast agents. In comparison with existing agents, gold provides greater X-ray attenuation per unit weight than iodine, it has favorable biocompatibility, and it has suitable chemical properties for bioconjugation [55].

Recent research efforts have been directed toward the application of biocompatible AuNPs as X-ray computed tomography contrast agents. In a recent study, unconjugated AuNPs were used as imaging agents for visualizing angiogenesis of tumor tissue. The researchers used synchrotron microradiology, microtomography and high-resolution X-ray microscopy as tools for visualization. The study demonstrated that bare AuNPs co-injected with heparin enabled visualization of the capillary bed and its leakage. It is evident that functional CT imaging of tumor microvasculature is possible by using AuNPs [56]. Further, dextran-coated AuNPs were used to detect atherosclerosis using X-ray CT [57]. In addition, dendrimer-coated AuNPs of sizes 2 to 4 nm, have demonstrated better CT contrast in the vascular system than the clinically available iohexol agent [58]. One commercial probe, AuroVist™ (Nanoprobes, Yaphank, NY, USA), is a water-soluble 15 nm AuNP designed for blood pool imaging. AuroVist™ demonstrates low toxicity and has sufficiently slow clearance (blood half-life = approx. 1 h) to enable *in vivo* imaging [59]. In this fashion, AuroVist™ aids researchers that lack the capability to perform continuous perfusion of iodinated reagents, or those that must utilize scanners that require longer acquisition times to achieve appreciable resolution. These attributes allow this gold nanomaterial to achieve much greater vascular contrast than an equivalent dose of an iodinated contrast agent. AuroVist™-enhanced CT imaging has also been combined with ultrasound imaging to develop computational flow dynamics simulations investigating the role of disturbed blood flow in the formation of abdominal aortic aneurysms [60].

#### 4.1.2. Example Data and Discussion

**Figure 4.**
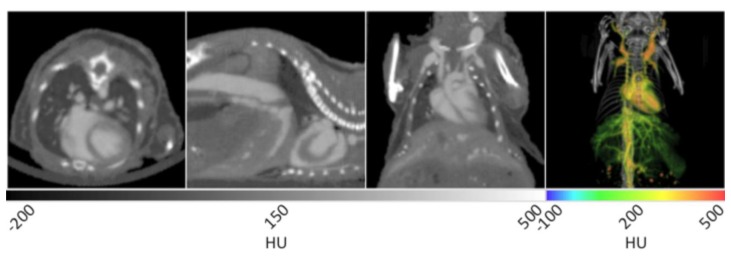
X-ray CT data of murine vasculature with AuroVist™ contrast enhancement. In the first three panels, the vasculature appears much brighter than the surrounding tissue due to the high concentration of gold nanoparticles in the blood. The contrast enhancement enables the visualization the heart, the aorta, and carotid arteries in addition to very fine vasculature in most organ systems. The right panel displays a 3D rendering in which the vasculature has been given a rainbow intensity scale to highlight its breadth in the heart, liver and kidneys.

The use of AuroVist™ enabled the imaging of vasculature within a number of organ systems when injected IV into a mouse (strain SKH1, male, 6 weeks old). As noted in Figure 4, major components of the cardiovascular system including the heart, carotid arteries, aorta, and hepatic vasculature are highlighted by the presence of the contrast agent. AuroVist™ provided adequate contrast enhancement of the heart to enable the visualization of even more detailed structures within it, including the aortic arch and individual chambers. In addition to the major components of the cardiovascular system, AuroVist™ also enabled the imaging of liver and renal vasculature, the iliac and femoral arteries, and the male reproductive organs (data not shown). In the first panel of Figure 4, the heart is clearly enhanced while the heart wall is visible as an area of darker contrast. In addition, an increase in contrast of the pulmonary vasculature was noted in the lungs. In the second panel from the left, the abdominal aorta, heart, heart wall, and a small amount of hepatic vasculature are highlighted by the AuNP. In the third panel, the heart, aortic arch, and carotid arteries are visible. The slice data in Figure 4 may be used to directly measure the diameter (µm) of vasculature at various anatomical locations, and also inspect for structural abnormalities. Unlike the figures illustrating natural lung tissue, adipose tissue, and brain tissue contrast, a VOI was drawn around vasculature and used to mask the surrounding skeletal structures. Once the skeletal structures with similarly high attenuation values were masked, the resulting image was then analyzed while retaining the original attenuation values for each voxel. The masked vascular data were given a rainbow intensity scale and overlaid on the original CT data to yield a 3D visualization of the mouse, given in the right panel of Figure 4. This 3D rendering shows all the previous structures in addition to extensive hepatic, spleen, and kidney vasculature. Quantitative data can be obtained for various regions of the vasculature through a ROI or VOI analysis of HU attenuation values in a given area. If a pre-injection CT image is obtained, it can serve as a control to determine the amount of contrast enhancement after various doses and time points.

Contrast enhanced microCT vascular imaging may provide valuable data in various models of clinical disease and in studies pertaining to the basic biology of underlying disease process. For example, tumor angiogenesis may be qualitatively assessed using contrast enhanced CT imaging [61]. Various quantitative measurements may be possible as well, including vascular dimension sizing [62]. Other researchers have evaluated vascular phenotypes of various mouse models with genotypes related to vasculature. The vascular phenotype was assessed for various anatomical regions using an X-ray contrast agent during planar imaging [63] that may be extrapolated to microCT. Of course, a specific protocol (appropriate imaging agent and imaging parameters) would need to be utilized to achieve experimental objectives. The specific range of applications for the vascular imaging protocol described here has not been specifically characterized; however, with various modifications and analysis optimization it is foreseeable that these methods may be applied to models of cardiac disease and tumor angiogenesis.

### 4.2. Liver

#### 4.2.1. Overview

The liver is prone to a wide range of common diseases. Liver cancer, especially hepatocellular carcinoma which accounts for approximately three-quarters of reported hepatic malignancies, caused an estimated 695,900 deaths worldwide in 2008 [64]. In addition, almost 32,000 deaths were attributed to cirrhosis in the United States in 2010 [36]. Mouse models have been developed for non-alcoholic liver disease [65], fibrosis [66], hepatitis C [67], and hepatocellular carcinoma [68] among others. MicroCT has been utilized to image liver tumors in mice with the aid of a hepatocyte specific contrast agent [69]. Contrast-enhanced studies have also proven the ability to detect the extent of hepatic fibrosis using an iodinated triglyceride (ITG) in analogous rat models of the disease [70]. This combination of high resolution CT imaging, contrast enhancement, and functional murine models of prevalent human diseases provides a powerful tool to aid in the understanding and treatment of liver diseases.

ExiTron™ nano 12000 (Miltenyi Biotec, Auburn, CA, USA) is a commercial contrast agent that enables CT imaging of the vasculature, liver, and spleen. ExiTron™ nano 12000 is an alkaline earth metal nanoparticle. Because alkaline earth metals exhibit high amounts of X-ray absorption, ExiTron™ provides excellent attenuation and contrast enhancement. After injection, ExiTron™ circulates in the blood stream which allows for vascular enhancement. Peak enhancement of the vasculature occurs at approximately 2 minutes post injection [71]. As the reagent circulates through the blood, it is taken up by Kupffer macrophages within the reticuloendothelial system in the liver, and peak contrast is observed 4 h post injection. Thus, the reagent is cleared through the liver and contrast enhanced regions represent areas of normal function. ExiTron™ nano 12000-enhanced CT imaging has also been used to update and improve the anatomical description of the murine liver and hepatic vasculature [72]. Contrast enhanced microCT has been used to image liver lesions, which will present as negative contrast due to a lack of functional uptake of contrast agent. For example, as the liver increases in density due to contrast enhancement by Fenestra™ VC [73], tumor metastases or other lesions are observed as darker contrast within the brighter tissue space [74]. The diameter (mm) and quantity of these cancer sites may be measured through straightforward image analysis. Unfortunately, while Fenestra™ VC was commercially available and widely used for microCT imaging of the liver, it was recently discontinued by the vendor (ART Advanced Research Technologies Inc., Montreal, QC, Canada). Another study involving liver metastases of colon carcinoma, found that contrast enhanced microCT imaging provided greater resolution than MRI with a fraction of the image acquisition time. MRI, however, did not require P.O. contrast enhancement, and the increased signal to noise ratio enabled the earlier detection of smaller lesions [9].

#### 4.2.2. Example Data and Discussion

**Figure 5.**
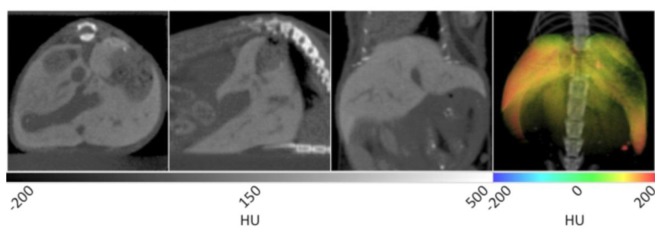
X-ray CT data of a murine liver with ExiTron™ nano 12000 contrast enhancement. In the first three panels, the liver tissue is distinguished from the surrounding tissues due to the uptake of contrast agent by Kupffer cells in the liver. A small amount of liver vasculature can be seen as negative contrast, especially in the coronal view in frame 3. The 3D rendering in the fourth panel shows the gross anatomical structure of the liver and spleen using a rainbow intensity map.

ExiTron™ was administered in daily aliquots of 25% of the recommended dose over a four-day period. This strategy was developed based on acute toxicity and mortality that was noted within our lab following the administration of a whole dose. This approach precluded imaging of the vasculature, but excellent liver and spleen contrast was observed due to high retention of the contrast agent therein. Figure 5 shows that liver tissue is greatly enhanced while fine liver vasculature may be distinguished via negative contrast.

In the transverse and sagittal planes, several lobes of the liver are enhanced while a small degree of vasculature is seen in negative contrast. In the coronal plane, a greater degree of fine vasculature can be seen in negative contrast along the periphery of the liver. It is on this bright palette that negative contrast arising from non-functional lesions may be observed as negative contrast. Spleen enhancement is also achieved to a similar degree as noted in the liver. In the 3D rendering at right, all lobes of the liver can be seen in addition to the spleen using a rainbow intensity scale generated in similar fashion to that described for vasculature. This image provides a view of the complete liver structure in three dimensions, in which gross anatomical changes may be visualized with disease progression.

### 4.3. Kidneys

#### 4.3.1. Overview

As of 2009, there were 398,861 end stage renal disease (ESRD) patients on some form of dialysis in the United States [75]. Further, the number of deaths attributed to various forms of kidney damage associated with kidney disease totaled over 50,000 in 2010, making kidney disease the eighth leading cause of death in the US [76]. With the aid of contrast enhancement, X-ray-CT imaging of the kidneys can provide immense anatomical data. Because many iodinated contrast agents undergo renal excretion, contrast enhancement of the kidneys is straightforward and will remain for relatively long time periods. Thus, abnormalities like renal cysts are readily detected and measured during longitudinal studies [77]. The availability of mouse models for renal diseases such as polycystic kidney disease (PKD) make microCT a feasible research tool for this and related disorders [78].

Iodixanol, more commonly referred to as Visipaque™ (GE Healthcare), is another widely used iodinated contrast agent. Visipaque™ is available in two concentrations, 270 mgI/mL and 320 mgI/mL. Varying the amount of iodine in the two products allows for different amounts of contrast enhancement per dose because the amount of X-ray absorption is proportional to the amount of iodine in the reagent. When Visipaque™ is present in high enough concentrations, the additional X-ray absorption of iodine allows for the contrast of perfused or targeted tissues. Visipaque™ can be injected intravenously via the retro-orbital plexus or through the tail vein with identical results. After injection, Visipaque™ circulates through the blood stream and is then filtered by the kidneys and eliminated through the bladder. Visipaque™ is the only commercially available iso-osmolar iodinated contrast agent. This property decreases the risk of nephrotoxicity compared to other iodinated compounds [79], and in clinical use, it enables 3D angiography by enhancing the X-ray attenuation of blood pool [80].

In research use, Visipaque™ enables the visualization of the kidneys and bladder during microCT, and allows accurate measurements of kidney volume (mm^3^), length (mm), and thickness (mm) [81]. Visipaque™ also has several possible applications outside of human or murine imaging. One prominent example reported the effectiveness of Visipaque™ as a contrast enhancement agent for the imaging of avian morphogenesis *in ovo* [82]. This technique enabled longitudinal CT imaging of live embryos after injecting a fertilized egg with Visipaque™. No adverse toxicity was reported. Visipaque™ can also be used as single sample method of determining glomular filtration rate (GFR) in research and clinical settings [83]. This method provides an advantage over the traditional method of measuring inulin clearance due to a decrease of stress related to the multi-sample method required for inulin measurement.

#### 4.3.2. Example Data and Discussion

**Figure 6.**
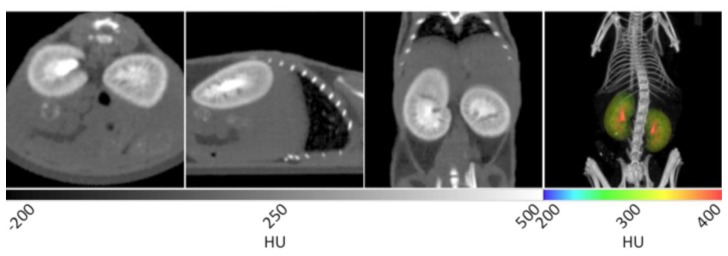
X-ray CT data of murine kidneys with Visipaque™ contrast enhancement. The renal cortex is noted along the outer rim of the kidneys due to its higher uptake than the adjacent medulla. Also, minor and major calyxes and the renal pelvis at the core can be seen due to the higher concentration of contrast agent in those collection ducts. In the 3D rendering at right, the renal cortex and medulla can be seen in yellow and green while the renal pelvis appears red due to its maximum concentration of Visipaque™.

With the aid of Visipaque™ 320, renal structures are greatly enhanced during CT imaging. In Figure 6, the renal pelvis (inner core structure) is immediately distinguishable from the renal cortex (outer rim) and medulla (intermediate space in between). In the sagittal plane, the cortex can be differentiated from the medulla by its higher degree of contrast enhancement. In addition, major and minor calyxes can be identified. Although not in the same plane as the kidneys, the bladder is also readily identified using Visipaque™ enhancement. For manual segmentation, the kidneys were separated into an independent data set by drawing a VOI around them and masking the surrounding tissue. This data set was then false colored using a rainbow scale, and overlaid onto the CT to yield the 3D visualization given in the right frame of Figure 6. The gross anatomical structure and positioning of the kidneys can be observed after the original CT image is overlaid to provide spatial reference points. The highly contrast-enhanced, red regions correspond to the renal pelvises, while the medulla and cortex are noted in yellow and green. Due to the high iodine content of Visipaque™ 320, such studies can be conducted with doses as low as 30 µL without sacrificing the diagnostic abilities of the reagent.

### 5.1. Gastrointestinal Tract

#### 5.1.1. Overview

As of 2004, inflammatory bowel diseases, mainly comprised of Crohn's disease and ulcerative colitis, affected approximately 1.4 million Americans [84]. MicroCT is capable of detecting the inflammatory response of the colon associated with colitis and Crohn's disease [85]. Further, microCT has demonstrated excellent capabilities for detection colon lesions in mouse models of cancer. Negative contrast-enhanced microCT imaging of murine models of colonic tumors has proven to be highly sensitive and specific for detecting lesions under 2 mm, validating the modality as a longitudinal imaging platform for investigating the disease [86,87]. Other studies have investigated the efficacy of multiple contrast-enhanced experiments for detecting colon malformations. A combination of intraperitoneal (IP) injected Iomeron^®^ with rectally administered Telebrix^®^, both iodinated contrast agents, was capable of detecting colon tumors [88]. Another method capable of detecting polyps in the colon requires the administration of barium followed by utilizing air as a negative contrast agent for the bowel space [89].

Barium sulfate is a contrast agent with a long history of use for both research and clinical imaging of the gastrointestinal tract. Barium is a heavy metal with high X-ray attenuation that enables it to provide great contrast in CT imaging. Unlike many barium solutions, BaSO_4_ is inert and largely non-toxic, and has a low frequency of adverse side effects [90]. In clinical use, BaSO_4_ is suspended and then ingested orally for upper-GI imaging, or a BaSO_4_ solution is administered rectally for lower GI imaging. Because BaSO_4_ is not digested or absorbed by any segment of the GI tract, it coats the lining of the esophagus, stomach, and intestines for a length of time sufficient for CT imaging. In clinical use, BaSO_4_ enhanced CT studies are commonly used to diagnose gastro-intestinal diseases such as Crohn's disease [91]. BaSO_4_ can also be used to image other abnormalities such as ulcers [92], post-inflammatory strictures, masses, fistulas, bowel wall edema associated with ischemia, and many other forms of gastrointestinal pathology.

#### 5.1.2. Example Data and Discussion

**Figure 7.**
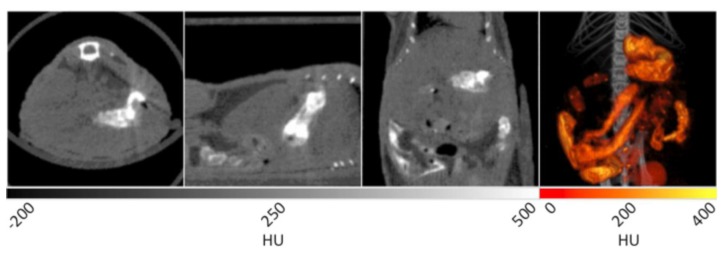
X-ray CT data of a murine gastrointestinal tract with barium sulfate contrast enhancement. After performing a best quality CT scan, the data was reconstructed with Albira software and analyzed via PMOD. In the first panel, the stomach can be seen and in the second and third panels, loops of the small intestine can be seen. The 3D reconstruction is false colored to aid in the visualization of the GI tract.

A mouse was fed a mixture of BaSO_4_ and peanut butter to facilitate ingestion for subsequent CT imaging. In Figure 7, several contrast enhanced components are observed. In the transverse plane, the stomach is noted as the bright tissue in the lower right quadrant of the image.

In the sagittal plane, the stomach is once again seen in the right hand side of the image. The coronal plane shows similar details of the GI tract as the sagittal plane, but with additional loops of the small intestine observed at bottom. The 3D image in the right panel displays the GI tract as a “fire” intensity scale overlaid on the original CT data set. This view provides the most compelling visualization of the architecture of the stomach and GI tract within the specimen. Varying the amount of time between ingestion of the reagent and performing the CT acquisition can allow for imaging of different regions of the GI tract. For example, if the data is acquired immediately after ingestion, the esophagus and stomach will be coated with BaSO_4_ to enable visualization, while the lower GI can be observed at later time points.

## 6. Contrast Agents in Research and Development Phase

At present, iodinated contrast media, along with barium sulfate suspensions in liquid media, are available for various clinical imaging purposes, while additional nanoparticle reagents are commercially available for preclinical use. Meanwhile, there are several new reagents and delivery techniques under investigation [55]. Iosimenol and GE-145 are both iodinated organic compounds derived from the structures of iodixanol and iotrolan, respectively [93,94]. Both exhibit lower osmolarity than the current clinically used compounds which enables the addition of electrolytes to the formulation to increase biocompatibility. In addition, iosimenol demonstrates lower viscosity than iodixanol which improves the ease of delivery. Dendrimer entrapped gold nanoparticles also show promise as a CT imaging contrast agent [95]. These AuNPs are highly stable through wide ranges of media, temperature, and pH, in addition to exhibiting improved X-ray attenuation over iodinated contrast media containing an equivalent molar concentration of radiopaque elements. Further, Nanoprobes Inc. has also demonstrated the potential of AuroVist™ for new applications in tumor imaging. While unconjugated AuroVist™ was successfully used to image brain tumors, it has also been conjugated to herceptin, a monoclonal antibody that targets the HER2 peptide found on aggressive forms of breast cancer, and shown to effectively image implanted tumors [96]. For a comprehensive review of contrast agents in development, the reader is directed to an excellent report from Lusic and Grinstaff [55].

## 7. Conclusions

Modern pre-clinical microCT technology enables high-resolution anatomical imaging for a variety of critical disease models in the soft tissue of living mice. The non-invasive nature of CT adds efficiency to longitudinal *in vivo* studies since multiple time points can be conducted on individual cohorts, thus reducing animal use and avoiding more time intensive experimental procedures. Using native contrast, tissues like lung, adipose, and brain can be readily distinguished from their surroundings. Diseases of the airway including cancer, emphysema, and fibrosis have been detected and quantified in mice using CT. The ability to visualize and measure visceral and subcutaneous adipose deposits over time also holds great potential. Brain tissue can be imaged and studied in models of cerebral ischemia. Other tissues rely on the aid of radiopaque contrast agents to enable their visualization. For imaging of the vasculature, many iodinated contrast agents provide extremely short-term blood pool imaging. Contrast agents, like iohexol, enable the direct visualization of cerebral vasculature to further enhance the study of diseases like stroke. Novel gold nanomaterials, however, reside in the blood for much longer periods of time and permit CT scans of the vasculature. This aids in the detection of AAA in addition to tumor angiogenesis through the enhancement of the rich vasculature therein. The commercially available contrast agent ExiTron™ nano 12000 facilitates contrast enhancement of the liver, which allows for the detection of liver lesions and fibrosis. The renal system is enhanced through the use of Visipaque™ to enable CT imaging of cysts in disease models of PKD and other abnormalities. Finally, barium sulfate can be ingested to enhance imaging of the gastrointestinal tract. This technique has been used for years in the diagnosis of GI disorders such as Crohn's disease. We anticipate that continued developments in the respective X-ray contrast agent and computed tomography instrument communities will permit imaging of new disease models with increased resolution and fidelity.

## Figures and Tables

**Table 1.** X-ray CT experimental methods.

	**Lungs**	**Fat**	**Brain**	**Vasculature**	**Liver**	**Kidneys**	**GI Tract**
*Animal*	SKH1, male, 6 wks old	B6.V-LepOB/J, male, 12 wks old	SKH1, male, 6 wks old	SKH1, male, 6 wks old	SKH1, male, 6 wks old Retro-orbital	SKH1, male, 6 wks old	SKH1, male, 6 wks old
*Contrast*	Native (air)	Native (fat)	Native (intracranium margin)	Retro-orbital 100 µL of 15 nm Aurovist (200 mg Au/mL)	100 µL 25% Exitron (diluted in saline) administered over 4 days	Retro-orbital 100 µL of Visipaque 320	BaSO_4_ mixed with peanut butter (50% by weight)
*Segmentation*	−500 to −200 HU (post masking outside thoracic region)	−200 to −50 HU (post masking outside body perimeter)	Masked to VOI	Masked to VOI	Masked to VOI	Masked to VOI	Masked to VOI
